# WAYFINDING DESIGN IN LONG-TERM-CARE COMMUNITIES: EVALUATION OF COMPLEXITY

**DOI:** 10.1093/geroni/igac059.2560

**Published:** 2022-12-20

**Authors:** Rebecca Davis, Margaret Calkins

**Affiliations:** Grand Valley State University, Grand Rapids, Michigan, United States; IDEAS Institute, Cleveland Hts, Ohio, United States

## Abstract

Wayfinding, the ability to find one’s way, is a significant problem for many older adults; especially those with cognitive impairment. Long term care communities (LTCC) are often fraught with challenges for wayfinding. Those who cannot find their way are at risk for decreased engagement and loss of independence. A critical need is to assess architectural and design features that promote effective wayfinding in an objective way. This study describes the results of a wayfinding design evaluation of 12 LTCC (4 Assisted living; 2 Independent Living; and 6 mixed residency). We measured space syntax axial integration (SSAI; a measure of the visual connectedness) and a Revised Wayfinding Checklist. The results showed low integration values in all communities, ranging from an R3 of 1.43-2.03, indicating low connectedness (and increased wayfinding complexity). The Checklist score totals ranged from 23 – 34 (M = 27.45) out of a possible total of 17 – 51. Results showed that the buildings were overall complex, all had long corridors (>100 feet), over half used multiple elevators to get to common areas; and over 60% had complex, multi-building layouts. Design wise, visibly accessible restrooms were not present; over half of the LTCC had insufficient lighting; and signage color/contrast and letter size was less than recommended. All sites had few directional signs at decision points. Thus, the review of these LTCC showed that most buildings were very complex, with low connectedness, and had room to improve design features like signage and lighting to support wayfinding ability for the residents.

